# Comparison of the recovery profile of sufentanil and remifentanil in total intravenous anesthesia: a systematic review and meta-analysis of randomized controlled trials^☆^

**DOI:** 10.1016/j.bjane.2024.844558

**Published:** 2024-09-07

**Authors:** Igor Seror Cuiabano, Rafael Pagliaro Naves, Rodrigo Bouchabki de A. Diehl

**Affiliations:** a Centro Universitário de Várzea Grande, Curso de Medicina, Várzea Grande, MT, Brazil; b Hospital de Cancer de Mato Grosso, Departamento de Anestesia, Cuiabá, MT, Brazil; c Hospital São Mateus, Departamento de Anestesia, Cuiabá, MT, Brazil

**Keywords:** Opioid Analgesics, Sufentanil, Remifentanil, Propofol, Intravenous anesthesia, Anesthesia recovery period

## Abstract

**Introduction:**

Remifentanil is a short-acting opioid and can be administered during surgery without the risk of delayed postoperative recovery but concerns about hyperalgesia and the shortages of remifentanil lead anesthetists to consider long-acting opioids for Total Intravenous Anesthesia (TIVA). Sufentanil is a more potent opioid with a longer context-sensitive half-life but can promote good postoperative analgesia due to its residual effect. This meta-analysis aimed to compare the recovery profile of remifentanil and sufentanil for TIVA.

**Methods:**

The search strategy was performed in PubMed, CENTRAL, and Web of Science for RCTs comparing sufentanil and remifentanil as part of TIVA in adults undergoing noncardiac surgery. Risk of bias and the quality of evidence were performed using RoB2 and GRADEpro, respectively. The primary outcome was time to tracheal extubation. Secondary analyses included postoperative analgesia, respiratory depression, and Postoperative Nausea and Vomiting (PONV).

**Results:**

Sufentanil increases the time to extubate, MD = 4.29 min; 95% CI: 2.33 to 6.26; *p* = 0.001. It also reduces the need for postoperative rescue analgesia, logOR = -1.07; 95% CI: -1.62 to -0.52; *p* = 0.005. There were no significant differences between both opioids for PONV, logOR = 0.50; 95% CI: -0.10 to 1.10; *p* = 0.10 and respiratory depression, logOR = 1.21; 95% CI: -0.42 to 2.84; *p* = 0.15.

**Conclusion:**

Sufentanil delays the time to tracheal extubation compared with remifentanil but is associated with a reduced need for postoperative rescue analgesia. No significant differences were observed between the two opioids in terms of postoperative respiratory depression or PONV.

## Introduction

Remifentanil is the most used opioid in the practice of Total Intravenous Anesthesia (TIVA).^1^ In addition to its strong synergy with propofol, remifentanil is characterized by a rapid onset and offset of action, facilitating titration, and allowing for long infusion times without delaying patient recovery.^2^ However, concerns regarding opioid-induced hyperalgesia and the limited availability of this opioid in certain regions have led anesthetists to explore alternative options to remifentanil. Sufentanil is currently considered the opioid with the highest potency, which is due to its pronounced affinity for µ-receptors.^3^ Compared to alfentanil, sufentanil has a shorter context-sensitive half-time for infusions of less than 8 hours.^4^ In addition, its residual effects may contribute to satisfactory postoperative analgesia, an aspect that does not occur with the use of remifentanil. In 2018, Wang et al published a meta-analysis comparing the efficacy and safety of sufentanil and remifentanil, particularly in patients undergoing craniotomy.^5^ While this study provided valuable insights, it was limited to a single type of surgical procedure. Therefore, we performed this meta-analysis to compare the recovery profile of TIVA with remifentanil and sufentanil in terms of time to tracheal extubation, postoperative pain, postoperative nausea and vomiting, and respiratory depression, considering a broader range of non-cardiac surgical procedures.

## Methods

The study was conducted by the recommended process of the Preferred Reporting Items for Systematic Reviews and Meta-Analyses guidelines (PRISMA).^6^ The protocol for this study was registered at PROSPERO under registration number CRD42022366691.

The research question was formulated using the PICOS (Population, Intervention, Comparator, Outcome Study) strategy: Does sufentanil delay tracheal extubation in adults undergoing non-cardiac surgery with TIVA compared to remifentanil? To address this, a systematic search was conducted in September 2022. For the PubMed and CENTRAL databases, a combination of MeSH terms and keywords including “remifentanil” and “sufentanil” were used, both as keywords ([All]) and as MeSH terms. In addition, “propofol” or “total intravenous anesthesia” were also searched for, again using keywords and MeSH terms. The strategy included terms related to randomized trials, using keywords such as “randomized” or “randomised” and the MeSH term “random”. The filters applied aimed to combine the anesthetics with randomized trials, resulting in the final search string reflecting the combination of all terms. For the Web of Science, the search strategy was carried out using text terms with the operator “All=”, which enables a broad search for any term in all fields of the article data. The terms used were “remifentanil”, “sufentanil”, “propofol” or “total intravenous anesthesia” as well as words related to randomization, such as “randomized” or “randomised”. The search was configured to identify studies that contained all the specified terms simultaneously using the final search string.

The results of the queries in the databases were imported into the Rayyan software ^7^ (https://www.rayyan.ai/) so that two authors (I.S.C. and R.P.N.) could search for titles and abstracts independently and in a blinded manner. Eligible studies for our review included indexed, published full articles of Randomized Clinical Trials (RCTs) that focused on adult patients undergoing non-cardiac surgery in which propofol-based anesthesia was used and in which the timing of extubation was compared between remifentanil and sufentanil. We specifically excluded studies that involved neuraxial blocks, intravenous adjuncts, inhalational anesthetics, or administration of sufentanil as an intermittent bolus. In addition, letters, editorials, and observational studies were excluded from our analysis. Data for this meta-analysis were extracted solely from peer-reviewed published articles. Data were collected using a standardized form. Information extracted included: author; age and sex of participants; number of members; type of procedure; duration of anesthesia; dose of sufentanil and remifentanil.

All analyses were carried out using STATA software (version 17). In some studies, outcome data were reported as median and interquartile range; therefore, we converted these data to mean and standard deviation using certain equations^8^^,^^9^ to summarize the effect estimate for all included studies. The random effects and fixed effects models were used when heterogeneity was present and absent, respectively. Heterogeneity was measured by the I^2^ statistic (> 50%) and the corresponding *p-*value (< 0.05). For continuous outcome variables (i.e., extubation time), the Mean Difference (MD) and the corresponding 95% Confidence Interval (95% CI) were pooled using the restricted maximum likelihood or inverse variance method if heterogeneity was present or absent, respectively. For dichotomous outcome variables (i.e., postoperative nausea/vomiting, postoperative rescue analgesia), the Mantel-Haenszel method was used to pool the log Odds Ratio (logOR) and the corresponding 95% CI across studies. If significant heterogeneity occurred in any of the analyses conducted, a leave-one-out sensitivity analysis was performed to determine whether the reported effect estimate would change after excluding one study at a time. Due to the small number of studies included (less than ten studies), an assessment of publication bias was not possible.

The Risk of Bias Tool^10^ developed by the Cochrane Collaboration was used to assess the methodological quality of each randomized trial. Two authors (R.P.N. and R.B.D.) independently reviewed and rated the elements of each trial using this tool. Data relevant to the analyses were extracted using this method. In case of discrepancies in the scoring or extraction of data, these were resolved by a third author (I.S.C.) through discussion. Finally, the quality of evidence for each outcome was evaluated by the same authors following the Grades of Recommendation, Assessment, Development, and Evaluation (GRADE) Working Group system^11^ using the GRADEpro Guideline Development Tool (https://www.gradepro.org/) to assess the certainty of the evidence and prepare the summary of findings.

## Results

**Figure 1:**
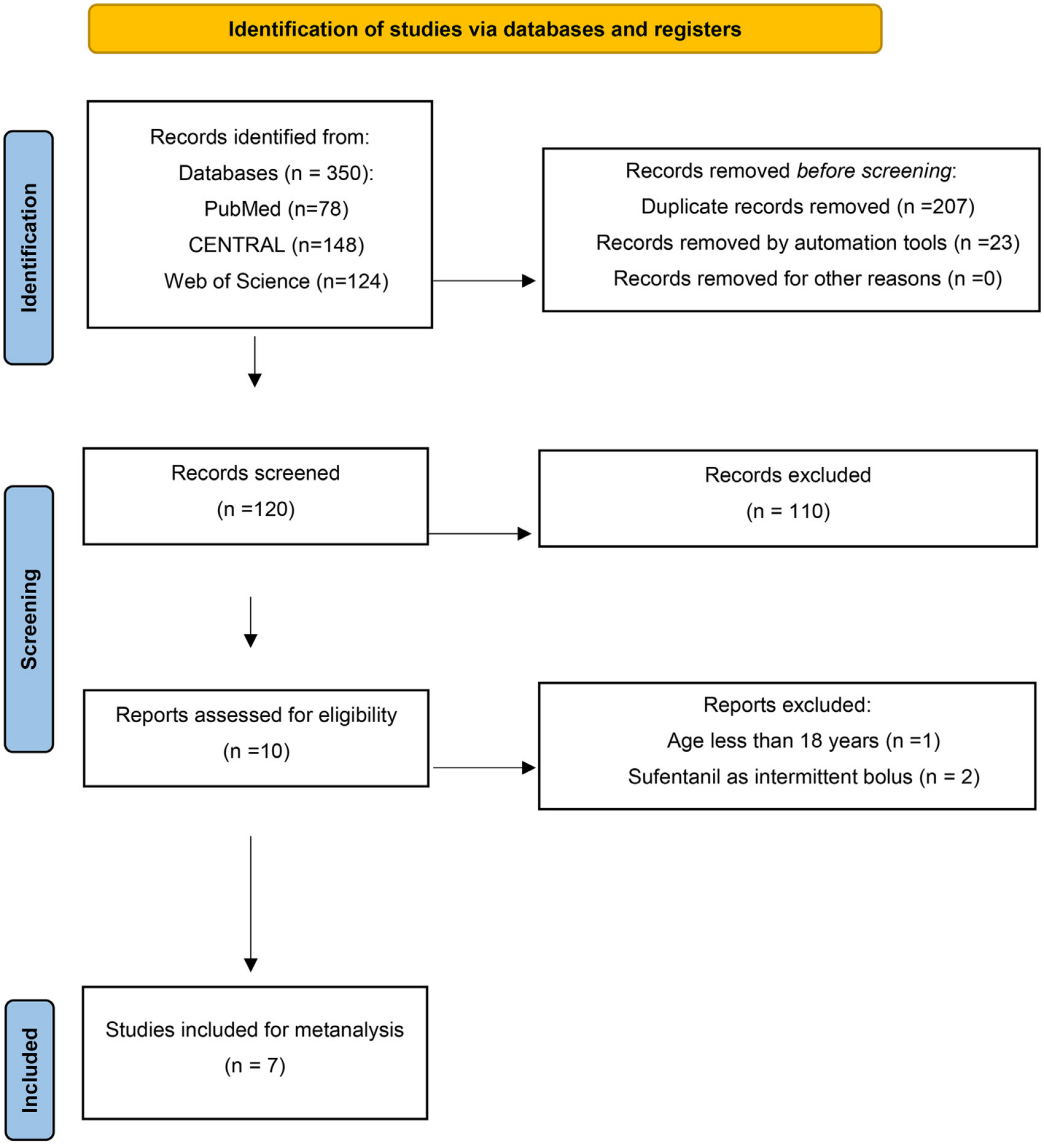
PRISMA flow diagram illustrating literature retrieved, included and excluded results from this review.

**Figure 2:**
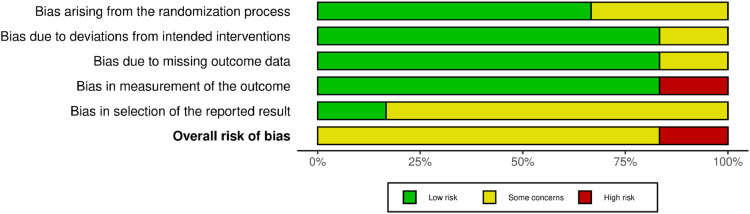
Risk of bias summary of the authors' assessments for each domain, expressed as percentages across all included studies.

Of the 350 studies identified in our literature search, seven met the inclusion criteria, involving a total of 403 patients (Fig. 1).^12^, ^13^, ^14^, ^15^, ^16^, ^17^, ^18^ The Cochrane Collaboration's Risk of Bias tool showed that most studies had a moderate risk of bias (Fig. 2). The type of surgery included craniotomy (three studies), intracranial surgery (one study), thyroid surgery (one study), colorectal surgery (one study) and non-gynecological surgery (one study). The absolute dose and administration approach of each procedure, as well as the baseline characteristics of the included trials, are summarized in Table 1.

**Table 1:** Characteristics of included studies.

Study	Surgical Procedure	Age (Mean ± SD)		Gender (Male)		Opioid Dose Regimen		Duration of anesthesia (min)	
				Sufentanil	Remifentanil				
		Sufentanil (n)	Remifentanil (n)	n (Total)	n (Total)	Sufentanil	Remifentanil	Sufentanil	Remifentanil
Billota et al.	Supratentorial craniotomy	54 ± 11	55 ± 14	14 (29)	13 (30)	Induced at 0.5 µg.kg^-1^ and then titrated to lower doses to maintain MAP within 10% of baseline	Induced at 0.5 µg.kg^-1^.min^-1^ and then titrated to lower doses to maintain MAP within 10% of baseline	273 (±58)	259 (±60)
Djian et al.	Nonemergency intracranial surgery	55.3 ± 9	45.7 ± 12	12 (31)	11 (29)	Induced at 0.25 µg.kg^-1^ and maintained at 0.0025 µg.kg^-1^.min^-1^	Induced at 1 µg.kg^-1^ and maintained at 0.25 µg.kg^-1^.min^-1^	251 (±92)	237 (±74)
Lentschener et al.	Thyroid surgery	48 ± 13	50 ± 15	3 (25)	8 (25)	An initial dose of 0.2 µg.kg^-1^ of sufentanil was given followed by a maintenance dose of 0.2 µg. kg^-1^.h^-1^	An initial dose of 1.5 µg.kg^-1^ was administered followed by a maintenance dose of 0.2 µg. kg^-1^.min^-1^	121 (±35)	110 (±26)
Liu et al.	Supratentorial craniotomy	44 ± 10	47 ± 13	15 (23)	12 (22)	TCI started with a Ce of 0.4 ng.mL^-1^ and then titrated according to hemodynamic changes between 0.2 and 0.4 ng.mL^-1^	For induction, TCI with a Ce of 5 ng.mL^-1^ and then titrated between 3 and 8 ng.mL^-1^ to maintain stable hemodynamics	277 (±27)	332.85 (±24.87)
Martorano et al.	Supratentorial craniotomy	52.8 ± 13	56 ± 13	15 (31)	20 (38)	Continuous infusion; dosage: 0.01‒0.04 µg.kg^-1^.min^-1^, reduced to 0.005‒0.020 µg.kg^-1^.min^-1^ after dura mater opening	Continuous infusion: dosage 0.2‒2 µg.kg^-1^.min^-1^, reduced to 0.1‒1 µg.kg^-1^.min^-1^ after opening the dura mater	280 (±17)	302 (±15)
Simoni et al.	Non-gynecological video laparoscopy	46 ± 12	42 ± 12	15 (30)	7 (30)	Induction consisted of a bolus of 0.5 µg.kg^-1^ followed by 0.5 µg.kg^-1^.h^-1^. If MAP increase, a 50 µg bolus was given. If MAP < 15% of baseline, the CI was turned off	Induction at 0.5 µg.kg^-1^.min^-1^ for 2 minutes and then 0.3 µg.kg^-1^.min^-1^ for maintenance. If the MAP > 15% of baseline, it was increased by 0.1 µg.kg^-1^.min^-1^	105 (±26)	95 (±9)
Vasian et al.	Colorectal surgery	61.4 ± 12	60.4 ± 11	20 (30)	19 (30)	TCI with initial Cp of 0.3 ng.mL^-1^ and then ranged between 0.2‒1 ng.mL^-1^ during the maintenance of anesthesia	TCI with Cp of 4 ng.mL^-1^ at induction, and between 3‒10 ng.mL^-1^ during maintenance of anesthesia	176.45 (± 63.7)	156.16 (± 30.1)

**Figure 3:**
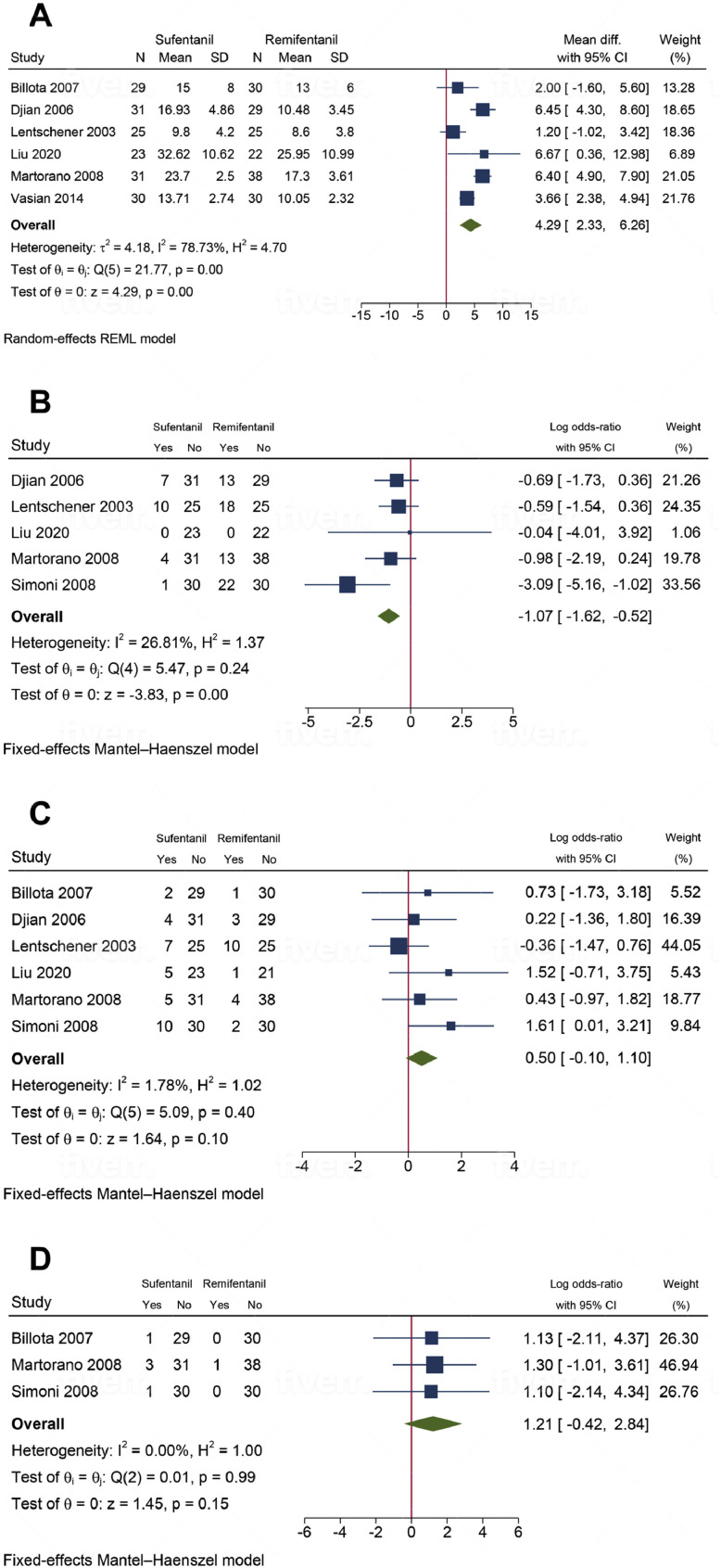
(A) Time to tracheal extubation was significantly longer in patients administered sufentanil compared to remifentanil. (B) The sufentanil group required significantly less postoperative rescue analgesics, indicating a lower need compared to the remifentanil group. (C) There were no significant differences in the incidence of PONV between the patient groups. (D) The incidence of respiratory depression was similar when sufentanil was compared to remifentanil in the setting of propofol-based TIVA.

**Figure 4:**
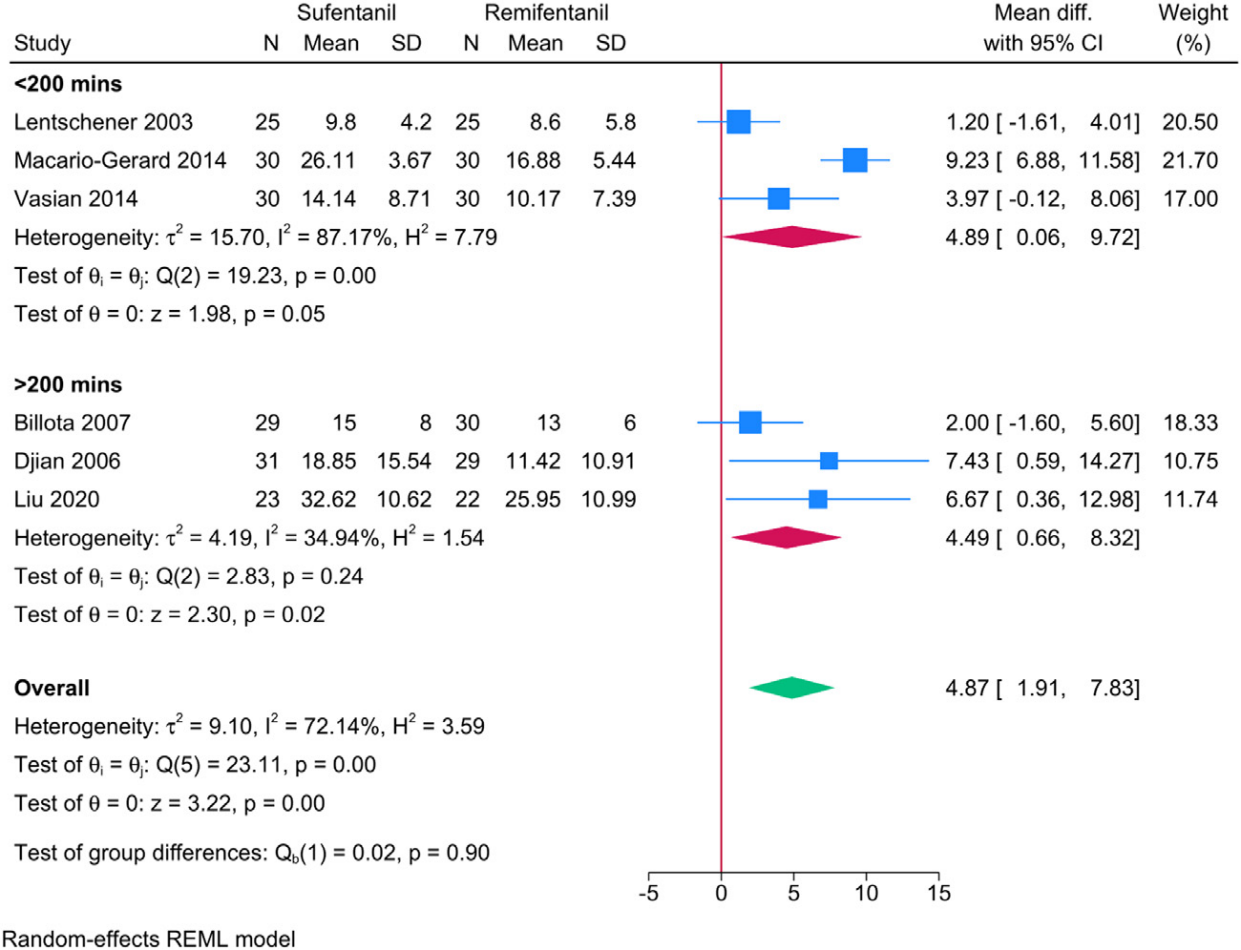
Subgroup analysis of tracheal extubation times under total intravenous anesthesia with remifentanil versus sufentanil, stratified by a 200-minute threshold. The analysis indicates reduced heterogeneity among patients receiving anesthesia for more than 200 minutes (I^2^ = 34.94%) compared to those receiving anesthesia for less than 200 minutes (I^2^ = 87.17%).

Tracheal extubation time was reported in the six trials.^12^, ^13^, ^14^^,^^16^, ^17^, ^18^ Overall, compared with remifentanil, patients who received sufentanil had a significant increase in the meantime to extubate (MD = 4.29 mins; 95% CI: 2.33‒6.26; *p* = 0.001) (Fig. 3A). However, we found significant statistical heterogeneity (I^2^ = 78.73%). Nevertheless, sensitivity analysis (leave-one-out) did not reveal any significant change in the reported effect estimate after excluding one study at a time. The subgroup analysis revealed that the duration of anesthesia was a contributor to heterogeneity (Fig. 4). For instance, the heterogeneity was resolved in patients receiving anesthesia for > 200 min (I^2^ = 34.94%, *p* = 0.24). Meanwhile, in those receiving < 200 min of anesthesia, heterogeneity remained substantial (I^2^ = 87.17%, *p* = 0.001). According to the GRADE system (Table 2), the evidence supporting our primary outcome was classified as low.

**Table 2:** Certainty of the evidence (GRADE).

Certainty assessment							N° of patients		Effect		Certainty	Importance
N° of studies	Study design	Risk of bias	Inconsistency	Indirectness	Imprecision	Other considerations	Sufentanil	Remifentanil	Relative (95% CI)	Absolute (95% CI)		
Extubation Time (assessed with: minutes)												
6	Randomized trials	Not serious	Serious^a^	Not serious	Not serious	Publication bias strongly suspected^b^	169	174	‒	MD **4.29 Min. higher** (2.33 higher to 6.26 higher)	⨁⨁○○ Low	Critical
Respiratory Depression												
3	Randomized trials	Not serious	Not serious	Not serious	Serious^c^	Publication bias strongly suspected^b^	5/95 (5.3%)	1/98 (1.0%)	**OR 1.21** (-0.42 to 2.84)	**2 more per 1.000** (from 15 fewer to 18 more)	⨁⨁○○ Low	Important
Postoperative Rescue Analgesia												
5	Randomized trials	Not serious	Not serious	Not serious	Not serious	Publication bias strongly suspected^b^	22/162 (13.6%)	66/210 (31.4%)	**OR -1.07** (-1.62 to -0.52)	**1.000 fewer per 1.000** (from 1.000 fewer to 627 fewer)	⨁⨁⨁○ Moderate	Important
Postoperative Nausea and Vomiting												
6	Randomized trials	Not serious	Not serious	Not serious	Serious^c^	Publication bias strongly suspected^b^	33/202 (16.3%)	21/194 (10.8%)	**OR 0.5** (-0.1 to 0.1)	**51 fewer per 1.000** (from 121 fewer to 96 fewer)	⨁⨁○○ Low	Important

CI, Confidence Interval; MD, Mean Difference; OR, Odds Ratio.

Explanations: (a) 78% heterogeneity; (b) less than ten studies were included in the meta-analysis, therefore it was not possible to assess publication bias; (c) Confidence interval includes the null value, it suggests significant uncertainty about the effect's direction and existence, indicating that observed effects could be due to chance, and necessitating cautious interpretation of these inconclusive results.

Postoperative rescue analgesia was assessed in five studies.^12^^,^^13^^,^^15^, ^16^, ^17^ Moderate evidence indicates sufentanil was associated with a significant reduction in the postoperative rescue analgesia as compared to remifentanil (logOR = -1.07; 95% CI -1.62‒-0.52; *p* = 0.005; I^2^ = 26.81%) (Fig. 3B). A total of six studies were conducted to examine the occurrence of PONV in patients.^12^, ^13^, ^14^, ^15^, ^16^, ^17^ Overall, no statistical differences for PONV were observed in patients that had received sufentanil compared to those who received remifentanil (logOR = 0.50; 95% CI: -0.10‒1.10; *p* = 0.10; I^2^ = 0%; low-certainty evidence) (Fig. 3C). Pooling data from three studies^14^^,^^15^^,^^17^ showed that there was no difference in the incidence of respiratory depression between sufentanil and remifentanil as part of propofol-based anesthesia (logOR = 1.21; 95% CI -0.42‒2.84; *p* = 0.15; I^2^ = 0%; low-certainty evidence) (Fig. 3D). For all the secondary outcomes, a subgroup analysis based on the assessment timepoint was not feasible due to the lack of relevant subgroups in at least two studies.

## Discussion

Our meta-analysis revealed a statistically significant increase in extubation time of approximately 4.29 minutes when using sufentanil compared to remifentanil for Total Intravenous Anesthesia (TIVA). Although the difference is statistically significant, its clinical relevance needs to be carefully assessed and contextualized, especially in major surgery. Postoperative pain management is critical in such procedures, and the potent analgesic effect of sufentanil may be beneficial. This meta-analysis indicates that using sufentanil is associated with substantial reduction in the need for postoperative rescue analgesia compared with remifentanil. This implies that the administration of remifentanil during surgery could potentially lead to higher postoperative opioid consumption, suggesting the possible development of Opioid-Induced Hyperalgesia (OIH).^1^ In situations where pain management is paramount, the advantages of sufentanil may outweigh the disadvantages of prolonged extubation. When choosing between the two drugs, the desired analgesia and the efficiency of extubation should be weighed.

Another important consideration is the risk of respiratory depression due to the accumulation of sufentanil after continued infusion. According to our review, although this finding is not significant, it can be attributed to the small sample sizes of the studies and can be considered relevant, although remifentanil requires the administration of long-acting opioid doses to ensure opioid analgesia, which is not free of complications such as respiratory depression.^19^ Given the accumulation of sufentanil with continuous infusion, perhaps the most appropriate approach to sufentanil-based anesthesia is to use its residual effect as the opioid element of a multimodal analgesic strategy. By avoiding combination with other long-acting opioids such as morphine, the aim is to achieve greater safety in terms of potential adverse effects. Even if sufentanil is discontinued before the end of the surgery, its effects may persist and affect respiratory dynamics. Clinicians should monitor respiratory parameters and be prepared to manage potential respiratory problems associated with prolonged sufentanil infusion.

The incidence of Postoperative Nausea and Vomiting (PONV) is remarkably high after surgical procedures. Studies have reported an incidence of 30% that can increase to as high as 80% in patients who are considered high-risk.^20^ Opioid analgesics remain the basis for the administration of general anesthesia, especially for TIVA, as propofol has no analgesic properties and the infusion of an opioid is necessary to ensure an adequate anesthetic level. In this review, we found that the incidence of PONV is similar with both opioids.

Interestingly, with the notable exception of postoperative respiratory depression, which was at higher risk with sufentanil, our results closely matched those reported by Wang in the context of craniotomy.^5^ The similarity of most of our results to Wang's study confirms the earlier findings in a broader surgical context and supports the generalizability of these results so that clinicians can extrapolate the data from craniotomy cases to other surgical scenarios, albeit with caution regarding respiratory side effects.

It is important to acknowledge that this review has several limitations. Among them is the lack of standardization of the technique of administering anesthetics. The differences in infusion techniques and dosages used in the various studies can be attributed to factors such as the variability in clinical practice, the individual characteristics of patients, the type of procedures performed and the specific objectives of each study. This heterogeneity contributes to a reduction in the certainty of evidence, as different opioid doses or methods of administration can significantly alter the depth and duration of anesthesia, as well as extubation time and associated side effect, highlighting the importance of future studies using more standardized methods.

Although most of the included studies used processed Electroencephalography (pEEG) monitoring to assist with propofol dosing to minimize the risk of overdose, it is important to note that such monitoring is not directly applicable to opioids. BIS is a valuable tool for assessing the effect of propofol on the level of hypnosis, but it does not directly indicate the intensity of pain,^21^ which would be beneficial for titrating opioid infusion, which can vary greatly from patient to patient and affects both the depth and duration of anesthesia.

In the current landscape of anesthesia care, there has been a marked shift from reliance on opioids to a more balanced approach known as “multimodal general anesthesia”. The rationale behind this approach is to use agents that act on different targets in the nociceptive system such as dexmedetomidine and non-specific agents such as magnesium, to ensure intraoperative control of nociception and postoperative pain management.^22^ The multimodal approach may significantly reduce the opioid doses required, as highlighted in previous studies. Such a strategy would likely have resulted in better postoperative pain control and reduced the need for rescue analgesics. In addition, time to extubation, a critical parameter in assessing recovery from anesthesia, may have been influenced by the opioid doses used. In the case of sufentanil, with its longer duration of action, the use of adjuvants could have allowed dose reduction, resulting in a more predictable and potentially shorter extubation time.

A notable strength of our meta-analysis is the exclusive inclusion of Randomized Clinical Trials (RCTs), often considered the gold standard in clinical research. However, a major limitation was that we were unable to conduct a funnel plot analysis due to the inclusion of less than 10 studies. The lack of funnel plot analysis leaves a gap in our assessment of publication bias.

In summary, this meta-analysis provides valuable insight into the comparative efficacy and safety of sufentanil and remifentanil in propofol-based intravenous anesthesia. It shows a shorter extubation time with remifentanil and better postoperative analgesia with sufentanil, although the lack of TCI pumps and different dosages protocols in manual infusion is a significant limitation. Future studies with larger sample sizes and standardized methodologies are crucial to validate and extend our results. Additionally, it would be beneficial to incorporate other variables of interest, such as long-term safety outcomes. Such studies will enhance our understanding of the long-term implications of the opioid selection under consideration and help in establishing more robust guidelines for clinical practice.

## Conflicts of interest

The authors declare no conflicts of interest.
